# Validity of the Japanese Version of the Weekly Calendar Planning Activity (WCPA)-10 on Assessing Executive Function in Patients with Acquired Brain Injury (ABI)

**DOI:** 10.3390/brainsci15090993

**Published:** 2025-09-15

**Authors:** Asako Matsubara, Yasuhiro Higashi, Mio Kawabata, Katsunobu Sugihara

**Affiliations:** 1 Department of Rehabilitation, Hiroshima City Rehabilitation Hospital, Hiroshima 7313168, Japan; 2 Department of Rehabilitation, Faculty of Health Science, Naragakuen University, Nara 6310003, Japan; y-higashi@naragakuen-u.jp; 3 Department of Rehabilitation Medicine, Hiroshima City Rehabilitation Hospital, Hiroshima 7313168, Japan; sugihara@soriha-hiroshima.jp

**Keywords:** functional cognition, executive functions, acquired brain injury, instrumental activities of daily living, neuropsychology, cultural adaptation, assessment

## Abstract

**Background**: The Weekly Calendar Planning Activity (WCPA) is one of the assessments of functional cognition, including executive function, by the cognitive instrumental activities of daily living (C-IADL). It is translated and adapted in many countries, but not in Japan. **Methods**: This study aimed to examine the validity of the Japanese version of the WCPA-10 (WCPA-10-J) in patients with acquired brain injury (ABI), and to explore the characteristics of cognitive strategy use among that population. Fifty-three patients with ABI aged 27–81 years and 52 healthy controls (HCs) completed the WCPA-10-J, three neuropsychological tests, and the assessment of the instrumental activities of daily living (IADL). **Results**: Results demonstrated that the WCPA-10-J was able to discriminate between the patients with ABI and the HCs. We found significant limitations in ABI patients’ ability to use strategies. Concurrent, convergent, and ecological validities were partly demonstrated through correlations between the neuropsychological test scores, IADL, and the WCPA-10-J performance. **Conclusions**: This study provides initial evidence for the validity of the WCPA-10-J for patients with ABI and suggests the need to use performance-based tests even in patients with normal cognitive screening test results. The WCPA-10-J could provide useful information for strategy-based interventions for patients with ABI.

## 1. Introduction

Acquired brain injury (ABI) is caused by sudden damage to the brain due to a variety of causes, including head trauma, stroke, brain tumors, and infections. Recent reviews have identified stroke and traumatic brain injury as the main causes of ABI [1]. In Japan, approximately 100,000 people die from stroke each year, making it the fourth leading cause of death among people. The Japan Stroke Association designates October as “Stroke Awareness Month” each year, promoting initiatives to raise awareness and prevent stroke (Ministry of Health, Labour and Welfare website, 2024). ABI can cause various impairments, but cognitive impairments in particular have a significant impact on daily life in the community.

Occupational therapists (OTs) are expected to be involved in improving occupational performance for those with cognitive deficits in the arena of cognitive rehabilitation. In OT professional journals, the first papers concerning cognition appeared in the middle of the 1960s, and the first official statement on cognition and occupational therapy was approved in the United States in 1991 [2,3]. According to Dr. Gillen’s Eleanor Clarke Slagle Lecture, “(OT involvement in cognitive rehabilitation) note the lack of early history and the early adoption of our colleagues’ approaches. Indeed, these times were turbulent, and science was advancing at a startling rate. We were unsure of our seemingly unsophisticated methods and adopted what appeared to be a more advanced and sophisticated approach. Our normalcy related to cognitive assessment can be described using the following descriptors: originally and primarily adopted from other disciplines, not occupation based, contrived, novel, two dimensional in a three-dimensional world, tested in an 8.5–11-inch space, and pen and paper or table top based.” [2] (p. 647). A similar situation continues even today with regard to the involvement of occupational therapists in cognitive rehabilitation in Japan.

In recent years, the term “functional cognition” has been the center of attention in the world of occupational therapy and has become a core concept in approaches to cognitive impairment, even though it is quite new for Japanese OT. It is believed that this term was first associated in occupational therapy with Claudia Allen’s cognitive disabilities model [4,5]. Functional cognition is defined as how people use and integrate their thinking and processing skills to accomplish everyday activities in clinical and community living settings [6] (pp. 1–25). It is also defined as the ability to use and integrate thinking and performance skills to accomplish essential ADLs and IADLs (Bar-Haim Erez & Katz, 2018; Giles et al., 2017; Wesson et al., 2016) [7,8,9]. In occupational therapy, functional cognition provides a core lens linking cognitive processes to real-world occupational performance.

Cognition and function have traditionally have studied separately due to the way various disciplines have developed their theories, conceptualizations, and measurement techniques [10,11]. However, it is necessary to assess cognition and function simultaneously because people, activities, and the environment are closely interrelated. Since “cognitive functioning can only be understood and facilitated fully within the context of occupational performance (everyday occupations such as self-care, home/community activities, education/work or study, and leisure)” [6] (pp. 1–25), it is strongly recommended that OT assess functional cognition using performance-based testing [4].

Allen was one of the first therapists to address the need to standardize observational cognitive assessment and established a hierarchy for classifying different levels of cognitive ability, known as the Allen Cognitive Level Scale [5,12]. She also developed a standardized screening tool of functional cognition—the Allen Cognitive Level Screen [13]. Moreover, other assessment tools, the Allen Diagnostic Module [14] and the Routine Task Inventory Expanded [15], were developed. And over time, several therapists have developed occupation-based functional–cognitive assessments, such as the Performance Assessment of Self-Care Skills (PASS) [16], the ADL-focused Occupation-Based Neurobehavioral Evaluation (A-ONE) [17,18], the Kitchen Task Assessment, and later, the Executive Function Performance Test (EFPT) [19].

The Weekly Calendar Planning Activity (WCPA) is based on the Dynamic Interactional Model of Cognition (DIC) and is one of the assessments of functional cognition. It is a new performance-based assessment of executive function (EF) that provides a broad analysis of how an individual manages and copes with a complex and cognitive instrumental activity of daily life (C-IADL) [20]. The WCPA has been used in various populations, including those with ABI [1,21,22,23], stroke [24,25], Parkinson’s disease [26,27], multiple sclerosis (MS) [28], attention-deficit hyperactivity disorder (ADHD) [29,30,31], epilepsy [32], mild cognitive impairment [33], schizophrenia [34,35], youth and adolescents at risk [36,37], neurodevelopmental or mental disorders [38], and healthy adolescents or older adults [39,40,41]. Previous research revealed good concurrent validity of the WCPA in patients with ABI [22], stroke [25], MC [28], ADAD [29], and good convergent validity [1], the high level of inter-rater reliability, and moderate to high test–retest reliability of the Chinese version of the WCPA (WCPA-C) [42]. The WCPA was translated into various languages and culturally adapted to Israel, Sweden, Finland, the Netherlands, Spain, and China. However, it has not been translated and examined in Japan. Furthermore, the WCPA has a short version (WCPA-10), which is easy to perform, with the advantages of being time-efficient and requiring minimal table space [25]. This feature makes it more suitable for the clinical environment and gives it the potential to be a valuable assessment for executive function in Japan.

The specific aims of this study were (1) to translate and adapt the WCPA-10 to assess executive function and refine it for the Japanese population, (2) to examine whether patients with ABI and healthy controls (HCs) would perform differently on the WCPA-10, and (3) to examine the relationships between performance on the WCPA-10 with neuropsychological tests and IADL assessment. It also aims to make a preliminarily exploration of the characteristics of cognitive strategy use in this population and its clinical application value in ABI patients by comparing the differences in the type and quantity of strategy use among the groups and examining the correlation between strategy use and other WCPA-10 valuables.

Guided by previous research on the WCPA and functional cognition, we specify the study hypotheses (H) and analytic plans as follows:

(H1) Group sensitivity: the WCPA-10-J will differentiate adults with ABI from healthy controls on core indices (accuracy, rules followed, error rates, number of strategies), with ABI expected to show lower accuracy/fewer rules and more errors.
(H2) Convergent/concurrent validity: We expected associations between WCPA-10-J accuracy and all external measures—MoCA-J, TMT-J A/B, FAB-J, and Lawton IADL-J—to reflect overlapping cognitive and functional demands. We did not prespecify relative magnitudes among measures; we estimate Spearman’s ρ with 95% confidence intervals and compare patterns descriptively.
(H3) Strategy use: compared with controls, adults with ABI will use fewer planning/monitoring strategies (e.g., written planning, fixed-then-flexible sequencing) during the WCPA-10-J.

## 2. Materials and Methods

### 2.1. Participation

The present study is a cross-sectional design. Fifty-three patients diagnosed with ABI by means of CT or MRI and 52 HCs were included in this study. Participants with ABI were recruited from people who received inpatient or outpatient (resident or ambulatory) rehabilitation at the Hiroshima City Rehabilitation Hospital, or at the Hiroshima City Daily Living Training Facility, from April 2025 to June 2025. The inclusion criteria for patients with ABI were (1) at least 18 years of age, (2) medically stable for assessment, and (3) having basic reading and writing skills. Exclusion criteria included those who had a history of other central nervous system diseases other than stroke, mental illness, current use of antipsychotic medications, and evidence of significant vision impairment and language problems (reading and writing alterations) that made it impossible to complete the test [25]. One patient was excluded due to a language problem.

Health controls were recruited using snowball sampling from people who worked in the hospital and the facility. Inclusion criteria were adults living independently in the community and with basic reading and writing skills in Japanese. Exclusion criteria were self-reported history of neurological disease, mental disorders, or use of antipsychotic medications [25]. This study was approved by the Ethics Committee Board of the Hiroshima City Rehabilitation Hospital (R67), and all the participants signed the informed consent.

### 2.2. Measures

For both patients with ABI and HCs, all measures were administered in a quiet room. We found a place for them where they could comfortably sit and write. The first author administered the WCPA, and the therapists responsible for the participants administered the other tests, including the TMT, FAB, MoCA, and the Lawton IADL scale. Participants were offered breaks as needed between tests throughout the assessment period.

#### 2.2.1. Weekly Calendar Planning Activity—Japanese Version-10 (Short Version, 10-Item; WCPA-10-J)

In this study, a short version of WCPA with 10 appointments was used, which is more feasible for the clinical setting as opposed to the 17-appointment original version [24]. With the original author’s permission, we followed a modified Brislin workflow (independent forward translations → reconciliation → independent back translation). Firstly, the original English text of WCPA-10 was translated into Japanese by two bilingual native researchers independently. Then, the two Japanese documents were compared with the original one, and the differences were discussed by the two translators until consensus was reached to form the first draft. The back translation was conducted by a translation specialist who was not involved in the first step. The back translation was reviewed and approved by the original WCPA author. Following her feedback, and after coordinating the opinions of the experts (OT, clinical psychologist, and physiatrist), we revised the first draft to form the second draft. Before data collection, we conducted cognitive debriefing with 4 healthy volunteers using think-aloud and structured probes to evaluate comprehension and ambiguity. Lastly, we made some cultural adaptations on appointments that are not suitable for the Japanese cultural context.

We replaced the appointments “Pick up medication at pharmacy” with “Pick up a birthday cake at the cake shop”, “Call to renew prescription” with “Call to book a birthday cake”, “Mail gas bill at post office” with “Pay apartment rent at the bank”, and “Call gas company to dispute late charge” with “Call the real estate company to confirm the apartment rent cost”, because these events are not very common activities for the Japanese, similar to the previous research [25]. Also, we adapted the expression of the date and time that may cause ambiguity (modifying the Japanese wording for “before” as “until” due to the grammatical characteristics of Japanese). In addition, the time system was adjusted from 12 to 24 h for all activities, and this change was also made in the Weekly Calendar sheet and the Weekly Calendar example sheet as well [1]. We received the feedback about these cultural adaptations from the original author of the WCPA, and the above translation process was completed with her consent.

During the test, participants were presented with a list of 10 appointments in random order and were required to enter them into a blank weekly calendar according to the five rules as follows: 1. No crossing out, 2. Tell me when it is (7 min after start), 3. No appointments/errands on a designated day, 4. Ignoring the 3 questions asked, 5. Tell the examiner when you are finished. It should be noted that some appointments are fixed (at a certain date/time) and others are flexible (can be entered at many possible dates/times, and may conflict with each other), Saturday and Sunday reversed, the calendar slots change, and the calendar ends at 8:30 p.m., but the appointment goes to 11:00 p.m. The examinees need to manage conflicts and enter appointments strategically. The rules and methods of entering appointments were explained orally to the examinee before the task. During the task, the examiner observed the examinee and recorded the type and number of strategies they used according to a list with 12 possible pre-identified strategies. The list of strategies included (1) underlines, circles, or highlights key words or features, (2) uses finger, (3) verbal rehearsal: repeats key words of instructions or appointment list out loud, (4) crosses off, checks off, or highlights appointments entered, (5) rearranges materials, (6) categorizes or organizes appointments before entering them (color codes, high lights, uses symbols, labels), (7) enters fixed appointments first, then flexible appointments, (8) written plan, (9) talks out loud about his or her strategy, method, or plan, (10) crosses off specified free day (Tuesday or Wednesday), (11) self-checks, and (12) pauses and rereads [20]. The main outcome measure included the number of entered appointments, accuracy (the number of correct items), total errors, total time for completion, planning time (from the start of the task to filling in the first appointment), the number and type of strategies used, the rules followed out of 5, and self-recognized errors [25]. The attention, including sustained attention (e.g., concentrate on the task and not distracted by three questions from the examiner), shifting attention (e.g., flexibility: if one strategy fails to accomplish the task, try another strategy), and divided attention (e.g., tell the examiner when it is, 7 min after start), is also checked in the testing.

#### 2.2.2. Montreal Cognitive Assessment—Japanese Version (MoCA-J)

The Montreal Cognitive Assessment (MoCA) is a cognitive screening tool for mild cognitive impairment (MCI) and for early detection of Alzheimer’s disease and dementia [43]. It assesses comprehensive cognitive domains, including attention, concentration, EF, memory, language, visuospatial skills, abstraction, calculation, and orientation. The total score of this test ranges from 0 to 30, with higher scores indicating better cognitive function. The Japanese version of the MoCA (MoCA-J) is widely used and effective for identifying MCI in elderly people with a cut-off score of 25 points [44].

#### 2.2.3. Trail Making Test—Japanese Version (TMT-J)

The Trail Making Test (TMT) [45] is one of the most commonly used neuropsychological tests of EF for patients with ABI in Japan. The TMT-J was standardized for healthy individuals aged 20 to 89. It is widely used as an evaluation method for individuals with higher brain dysfunction due to traumatic brain injury, mild cognitive impairment, and relatively mild dementia, and relatively pure executive function disorders represented by frontal lobe damage. The TMT-J part A (TMT-J-A) requires participants to draw a line and connect 25 numbers (1–25) randomly distributed on a piece of paper. The TMT-J part B (TMT-J-B) consists of both numbers (1–13) and letters (あ-し), which asks the participants to draw a line alternating between numbers and letters (e.g., 1-あ-2-い-3-う), the same as the TMT-B. The subjects were asked to complete the test. Before the test begins, there are templates for subjects to practice. The time required to complete the two tasks was recorded. The less time required means better performance. A maximum of 5 min (300 s) was allowed for the test. The whole process was observed closely to detect errors as soon as they were made, and immediate feedback was provided.

#### 2.2.4. Frontal Assessment Battery—Japanese Version (FAB-J) [46]

The Frontal Assessment Battery (FAB) evaluates frontal lobe function. It consists of six items that examine language conceptualization (understanding similarities), language fluency, motor programming, sensitivity to interference, inhibitory control, and comprehension behavior [47]. It takes approximately 10 min to administer. The lower the score, the higher the possibility of frontal lobe dysfunction.

#### 2.2.5. Lawton Instrumental Activity of Daily Living (IADL) Scale—Japanese Version (Lawton IADL-J)

The Lawton IADL-J was adapted from the work of Lawton and Brody [48], which is a self-reported IADL measurement tool for older adults, by the Japan Geriatrics Society. The Lawton IADL-J is one of the most common IADL scales in Japan. The contents of the Lawton IADL-J included eight items of IADL tasks, including the use of the telephone, shopping, meal preparation, housework, laundry, transportation, medication management, and financial management. These items consist of 3 to 5 subitems. The scores of the Lawton IADL-J range from 0 to 8, and a higher score represents better performance in the IADL.

### 2.3. Statistical Analysis

The normality of continuous variables was assessed using the Shapiro–Wilk test and visual inspection of Q–Q plots. When normality was violated, we used Mann–Whitney U tests (two-tailed) for continuous variables, χ^2^ tests or Fisher’s exact tests for categorical variables, and Spearman’s ρ for associations. We report effect sizes (e.g., r = |Z|/√N) and 95% CIs when feasible. For 12 strategy–frequency comparisons, we applied Bonferroni correction (α = 0.05/12 = 0.004). Between-group differences (ABI and HC) in continuous variables were analyzed using the Mann–Whitney U test.

Associations between the WCPA-10-J variables (e.g., accuracy, number of strategies used, number of errors) and neuropsychological test scores (MOCA-J, TMT-J-A, TMT-J-B, FAB-J), as well as Lawton IADL-J, were examined using Spearman’s rank-order correlation coefficients. The significance level was set at *p* < 0.05. We used the Chi-square test to compare the frequency of strategies used between the two groups. We also carried out a power analysis with G*Power software (ver. 3.1.9.7; Heinrich-Heine-Universität Düsseldorf, Düsseldorf, Germany) to confirm that the statistical power was sufficient. Based on an estimated medium effect size (Cohen’s d = 0.5), the sample size of 53 patients and 52 HC provided sufficient statistical power (1 − β ≈ 0.79) to detect significant group differences at an alpha level of 0.05. We used the SPSS Statistics version 30 (IBM Corp., Armonk, NY, USA) for statistical analyses.

## 3. Results

Table 1 shows the demographics and clinical characteristics of all subjects in this study.

### 3.1. Difference Between the ABI Group and the HC Group on the WCPA-10-J (Table 2)

Due to violations of normality assumptions, nonparametric comparisons were conducted using the Mann–Whitney U test. Results revealed that the stroke group demonstrated significantly lower performance than the HC group in terms of WCPA-10-J accuracy (U = 483.5, Z = −5.89, *p* < 0.001), number of cognitive strategies used (U = 689.0, Z = −4.47, *p* < 0.001), number of rules followed (U = 689.5, Z = −4.95, *p* < 0.001), and made more errors (U = 523.0, Z = −5.74, *p* < 0.001). No significant group differences were observed for total time (*p* = 0.080), number of entered appointments (*p* = 0.119), or self-corrected errors (*p* = 0.062), although a trend toward significance was noted for total time and self-corrections.

### 3.2. Correlations Between the WCPA-10-J, Neuropsychological Assessments, and IADL (Table 3)

Spearman’s rank-order correlation analysis showed that WCPA-10-J accuracy was positively correlated with the MoCA-J (ρ = 0.576, *p* < 0.01), and negatively correlated with the TMT-J-A (ρ = −0.449, *p* < 0.01) and the TMT-J-B (ρ = −0.556, *p* < 0.01). The number of cognitive strategies used was negatively correlated with the TMT-J-B (ρ = −0.337, *p* < 0.05) and positively with total completion time (ρ = 0.313, *p* < 0.05). Errors were negatively correlated with the WCPA-10-J accuracy (ρ = −0.821, *p* < 0.01) and positively with the TMT-J-A (ρ = 0.428, *p* < 0.01) and the TMT-J-B (ρ = 0.430, *p* < 0.01). the Lawton IADL-J scores were positively correlated with the WCPA-10-J accuracy (ρ = 0.350, *p* < 0.05) and negatively with the total errors (ρ = −0.314, *p* < 0.05).

### 3.3. Types of Strategies, Correlations Between WCPA-10-J Strategies Used and Other Variables (Table 4 and Table 5)

There are statistically significant differences in the type of strategies, including rearrangement of materials, categorizing or codes, fixed appointments first, written plan, and crossing off specified free day, which were used to complete the task between the two groups. Among all participants (*n* = 105), the WCPA-10-J accuracy and total errors had significant moderate relationships with the WCPA-10-J strategies used. In the HC group, strategy use was positively correlated with the WCPA-10-J total time and negatively correlated with total errors.

## 4. Discussion

This study aimed to translate and adapt the WCPA-10 to assess executive function and refine it for the Japanese population, and to initially verify the validity of the WCPA-10-J in adults with ABI.

### 4.1. Difference Between the ABI Group and the HC Group on the WCPA-10-J

Our results demonstrated that patients with ABI performed significantly worse than HCs on the WCPA-10-J variables, including accuracy, strategies used, rules followed, and total errors, which were the same or partly similar to the previous study [1,24,25]. The WCPA-10-J was able to discriminate between patients with ABI and the HCs. This finding highlights the possible value of the WCPA-10-J in patients with ABI who perform normally on cognitive screening tests to detect potential subtle EF difficulties that may affect daily functioning, similar to other versions of WCPA. Even though more than half the subjects in our study achieved a high score in the MoCA-J, some of them showed WCPA-10-J performance deficits, in the same way as the previous study suggested [24]. It is also suggested that the WCPA-10-J may assist in identifying patients in need of comprehensive evaluation. We used screening tests in this study, so we need to use detailed neuropsychological tests to clarify the relationship between the performance deficits and the specific cognitive impairment in the EF in future studies.

Unexpectedly, both the ABI patient group and the HC group performed similarly in planning time and total time for completion, supporting the previous study [1,25]. These two variables seem to be associated with personal factors such as habits, patterns, and the types of strategies used for completing this task [24]. Previous studies reported that some participants who spent a much longer time planning using written plans were more often in the control group, and some participants spent more time on reading, understanding, and entering the appointments were in the patient group [25]. Our results support this, and the HCs spent a longer time planning using the strategy of written plans. In this study, we did not find any statistically significant differences in time use, including the total time and the planning time, between the two groups. Moreover, both the patients and HCs in this study took longer to complete the WCPA-10-J than the subjects of the previous studies [1,24], similar to another previous study [25]. Two mechanisms may underlie this pattern. First, a speed–accuracy trade-off: HCs often invested time in pre-planning and self-checks, which increased the time but reduced errors. Second, the strategy selection: in our sample, strategy use correlated positively with total time and negatively with errors in HCs, suggesting longer time reflects deliberate monitoring rather than inefficiency. These mechanisms compress raw time differences while preserving clear group differences in accuracy, rules, and errors. In addition, several Japanese cultural tendencies may help explain cross-national differences. (i) Error-avoidance and precision norms can promote drafting and self-checking, increasing total time while reducing errors (particularly in HCs). (ii) Planning style: written planning, categorization, and fixed-then-flexible sequencing are often seen as “doing it properly,” yet patients with ABI used these strategies less. (iii) Temporal and task conventions: adopting a 24 h time format and appointments (e.g., rent payment, birthday-cake ordering) reduces unfamiliarity, but rule anomalies (e.g., weekend reversal) still tax executive control. These may explain longer times yet fewer errors in HCs and strategy-related vulnerabilities in ABI. In this study, almost half of the HCs made a rough draft first, and more than half provided the self-check at the end of the task. This still needs to be explored in association with cultural features in further studies.

### 4.2. Correlations Between the WCPA-10-J, Neuropsychological Assessments, and IADL

Our major finding was that the WCPA-10-J accuracy and total errors were significantly associated with TMT-J-A, TMT-J-B, and MoCA-J, which provided support for the concurrent validity of WCPA-J-10. The correlations were moderate, which was consistent with the previous validity study on the WCPA in ABI [22], stroke [25], Parkinson’s disease [26], and multiple sclerosis [28]. It was suggested that the WCPA may overlap only partially with traditional neuropsychological tests. The number of variables in the WCPA-J-10 associated with traditional neuropsychological tests was lower than the results of previous studies. The cognitive status of subjects: more than half of ABI patients were classified as cognitively normal according to MoCA-J in this study, may affect this result. It also suggests that several Japan-relevant tendencies of strategy-use differences may help explain cross-national differences in the results of the WCPA-10-J, such as “error-avoidance (e.g., drafting and self-checking)” and “planning style (e.g., written planning, categorization, and fixed-then-flexible sequencing)”. We need to examine this in a future study. It was also found that the WCPA-10-J variables were more strongly correlated with the MoCA in this study, the same as in the previous study [25]. This may be because the MoCA, which covers nine different cognitive subdomains related mainly to frontal lobe functions, reflects cognitive abilities more comprehensively. Moreover, we found the WCPA-10-J variables were slightly more strongly correlated with TMT-J-B than with TMT-J-A. It is suggested that TMT-B is more complex and harder than TMT-A and requires more executive control [49], and the relative complexity and challenge of the WCPA-10-J [25].

Lawton IADL-J correlations with WCPA-10-J were modest yet statistically significant (accuracy ρ = 0.35; errors ρ = −0.31; *p* < 0.05), whereas FAB-J associations were weak/non-significant in this study. Attenuation likely reflects construct differences (it may be because the FAB is one of the screening tests for assessing frontal lobe function, and samples broad frontal behaviors with a limited range vs. WCPA-10-J’s multistep planning/error management, or because of the ceiling effect) and method differences (Lawton is self/proxy-report and confounded by physical status [25]; WCPA-10-J is performance-based). In addition, a limited setting, including inpatient or residential settings in this study, or a non-observable aspect of this assessment, may affect our results. The WCPA requires multitasking, planning, and inhibitory control, and has the potential to reflect well on the individual’s performance in completing complex tasks in everyday life [25]. Future studies should pair WCPA-10-J with performance-based IADL (e.g., EFPT, PASS) and domain-specific executive tests.

### 4.3. Types of Strategies, Correlations Between WCPA-10-J Strategies Used and Other Variables

Our results demonstrated that the WCPA-10-J accuracy and total errors had significant moderate relationships with the strategies used by all participants. And, strategy use was positively correlated with the WCPA-10-J total time and negatively correlated with total errors in the HC group. These findings support the suggestion that effective strategy use helps individuals acquire and process information and promote the efficient utilization and allocation of cognitive resources [50]. The patients with ABI used significantly fewer strategies to complete the task than the HCs, but a higher number than those used in previous studies [24,25]. Our results support the suggestion that impaired self-awareness may help explain why patients with stroke use fewer strategies [51,52].

It is an interesting finding that the types of strategies used to complete the task in this study were partly different from those used in previous studies [1,24,25]. Specifically, the patients with ABI statistically used the strategies less, including “re-arrangement of materials”, “entered fixed appointments first, then flexible”, “uses written plan, makes a rough draft first, or plans”, and “cross off specified free day” in this study. It is also interesting that almost all of these strategies belong to the ‘Simplifies or Organizes’ category, and this may be related to the cultural aspect. We also need to examine the association between the strategy used and the cultural aspect in future studies.

It is believed that cognitive strategies are commonly thought of as compensation to improve patients’ ability to function in their daily lives. The previous studies reported the association between strategy use and functional outcome in patients with ABI [53,54]. The WCPA can provide valuable information for interventions, such as the objective quantification of the number and type of strategies used, which might provide a basis for goal setting and training during tailored interventions [25]. Our results indicate that the WCPA-10-J can also provide valuable information for OTs to set goals and to make unique interventions for all our clients in Japan.

The present study has several limitations. This study was conducted at a single urban hospital and one training facility, which may limit generalizability. Regional variation in clinical practice (e.g., intensity and structure of cognitive rehabilitation), referral patterns, and patient demographics (urban vs. non-urban) could influence WCPA-10-J performance. Therefore, caution is warranted when extrapolating to broader Japanese or international ABI populations, and multi-center studies are needed. Our clinical characteristics of ABI are relatively limited, because this study was conducted in one hospital and facility. Also, we need to consider more comprehensive clinical information, such as ABI severity, lesion-specific location and size, or comorbidities. Future studies should further explore the relationship between clinical characteristics and performance on the WCPA-10-J. More than half of ABI patients were classified as cognitively normal according to MoCA-J in this study. Future studies with larger samples are needed and should also examine a wider range of variations in levels of cognitive impairments. As mentioned above, we need to examine the ecological validity of using the performance-based IADL assessment.

## 5. Conclusions

This study provides initial evidence for the validity of the WCPA-10-J for patients with ABI. The results of this study suggest the need to use the performance-based test even in patients who perform normally on cognitive screening tests to detect potential difficulties that may affect daily functioning. Based on the WCPA-10-J performance, we found significant limitations in ABI patients’ ability to use cognitive strategies, which were also associated with impaired task performance. The WCPA-10-J has the potential to provide actionable strategy and error profiles. Goal setting: when written planning or fixed-then-flexible sequencing is absent, target a preview–mark conflicts–draft–enter–self-check routine. Strategy training: implement the multicontext approach to strengthen online awareness and self-monitoring (e.g., crossing off entered items, verbal rehearsal, scheduled self-checks). Environmental supports: use color-coding, category grouping, and salient rule cues (e.g., no appointment on Wednesday). Interdisciplinary planning: align discharge tasks (medication renewal, bill payment, appointment management) with graded executive demands based on WCPA-10-J profiles.

## Figures and Tables

**Table 1:** Demographic and clinical characteristics of patients with stroke and HCs.

	ABI (*n* = 53)	HCs (*n* = 52)
Age	54.7 (13.6)	49.0 (10.2)
Gender		
Males	39 (73.6%)	25 (48.1%)
Females	14 (26.4%)	27 (51.9%)
ABI type		
Stroke (Ischemic)	18 (33.9%)	
Stroke (Hemorrhage)	30 (56.6%)	
Traumatic subarachnoid hemorrhage	3 (5.7%)	
Encephalitis	1 (1.9%)	
Post-resuscitation encephalopathy	1 (1.9%)	
Brain location		
Lef hemisphere	24 (45.3%)	
Right hemisphere	20 (37.7%)	
Bilateral	7 (13.2%)	
Cerebellum	2 (3.8%)	
Time after onset		
within 2 weeks	1 (1.9%)	
within 6 months	31 (58.5%)	
over 6 months	21 (39.6%)	

**Table 2:** Comparison between groups of performance on the WCPA-10-J and neuropsychological tests (mean ± SD).

WCPA-10 Measure	ABI (*n* = 53)	HCs (*n* = 52)	Mann–Whitney U	Z	*p*-Value (2-Tailed)
Total time (s)	1020.4 (509.7)	900.8 (443.2)	1082.5	−1.752	0.080
Planning Time (s)	182.3 (345.3)	295.8 (805.2)	1487.5	0.702	0.483
Entered appointments	9.6 (0.8)	9.8 (0.4)	1226.0	−1.560	0.119
Accuracy *	7.4 (1.5)	9.1 (0.9)	483.5	−5.886	<0.001
Strategies used *	4.2 (1.8)	6.1 (2.2)	689.0	−4.471	<0.001
Rule followed *	4.0 (0.8)	4.7 (0.4)	689.5	−4.953	<0.001
Total errors *	1.8 (1.3)	0.4 (0.6)	523.0	−5.736	<0.001
Self-recognized errors	0.3 (0.4)	0.2 (0.5)	1157.5	−1.864	0.062

* *p* < 0.05 was considered statistically significant.

**Table 3:** Correlations between the WCPA-10-J, neuropsychological test, and IADL scale only for the patients with ABI (*n* = 53).

WCPA-10 Measure	Entered Appointments	Accuracy	Total Errors	Total Time	Planning Time	Rule Followed	Strategy Used	Self-Recognized Errors
TMT-J-A	−0.20	−0.45 **	0.43 **	0.32 *	0.11	−0.18	−0.14	−0.06
TMT-J-B	−0.32 *	−0.56 **	0.43 **	0.28 *	0.14	−0.24	−0.34 *	−0.09
FAB-J	0.09	0.18	−0.14	−0.22	−0.18	0.13	0.11	−0.05
MoCA-J	0.15	0.58 **	−0.49 **	−0.22	−0.02	0.17	0.21	0.01
Lawton IADL-J	0.14	0.35 *	−0.31 *	0.01	0.14	0.14	0.22	0.05

* *p* < 0.05, ** *p* < 0.01.

**Table 4:** Comparison of the type and frequency of strategies used.

Strategy	ABI (*n* = 53) *n* (%)	HCs (*n* = 52) *n* (%)	Pearson χ^2^	*p*-Value
Highlights	21 (40)	27 (51)	0.95	0.3292
Uses finger	45 (85)	45 (85)	0	1
Verbal rehearsal	37 (70)	32 (60)	0.66	0.415
Crosses off/checks off	40 (75)	41 (77)	0	1
Rearrangement of materials *	6 (11)	16 (30)	4.65	0.0311
Categorizes or codes *	11 (21)	17 (32)	1.21	0.2707
Fixed appointments first *	14 (26)	32 (60)	11.1	0.0009
Written plan *	7 (13)	26 (49)	14.26	0.0002
Talks aloud	8 (15)	6 (11)	0.08	0.7742
Crosses off specified free day *	5 (9)	18 (34)	8	0.0047
Self-checks	23 (43)	32 (60)	2.42	0.1199
Pauses and rereads	7 (13)	11 (21)	0.6	0.4377

* Bonferroni correction was applied for multiple comparisons across 12 strategies. The adjusted significance level was set at *p* ≤ 0.004.

**Table 5:** Correlations between the strategy used and other WCPA-10-J variables.

	Strategies Used		
	ABI (*n* = 53)	HCs (*n* = 52)	All Participants (*n* = 105)
Accuracy	0.174	0.309 *	0.432 **
Entered appointments	0.118	0.147	0.187
Total errors	−0.077	−0.429 **	−0.408 **
Total time	0.290 *	0.466 **	0.249 *
Planning Time	0.245	0.328 *	0.229 *

* *p* < 0.05, ** *p* < 0.01.

## Data Availability

The data presented in this study are available on request from the corresponding author due to ethical reasons.
